# Targeting Myeloid-Derived Suppressor Cells Derived From Surgical Stress: The Key to Prevent Post-surgical Metastasis

**DOI:** 10.3389/fsurg.2021.783218

**Published:** 2021-12-09

**Authors:** Sha Zhu, Yunuo Zhao, Yuxin Quan, Xuelei Ma

**Affiliations:** ^1^Department of Urology, Institute of Urology, West China Hospital, Sichuan University, Chengdu, China; ^2^Department of Biotherapy, Cancer Center, West China Hospital, Sichuan University, Chengdu, China; ^3^State Key Laboratory of Biotherapy, Department of Biotherapy, Cancer Center, West China Hospital, Sichuan University, Chengdu, China; ^4^West China School of Medicine, West China Hospital, Sichuan University, Chengdu, China

**Keywords:** myeloid-derived suppressor cells, stress, surgery, tumor recurrence, metastasis

## Abstract

Myeloid-derived suppressor cells (MDSCs) are known to play an essential part in tumor progression under chronic stress settings through their manipulation of adaptive and innate immune systems. Previous researches mainly focus on MDSC's role in the chronic tumor immune environment. In addition, surgery can also serve as a form of acute stress within the patient's internal environment. Nevertheless, the part that MDSCs play in post-surgical tumor development has not gained enough attention yet. Although surgery is known to be an effective definite treatment for most localized solid tumors, there are still plenty of cancer patients who experience recurrence or metastasis after radical resection of the primary tumor. It is believed that surgery has the paradoxical capability to enhance tumor growth. Many possible mechanisms exist for explaining post-surgical metastasis. We hypothesize that surgical resection of the primary tumor can also facilitate the expansion of MDSCs and their pro-tumor role since these surgery-induced MDSCs can prepare the pre-metastatic niche (the “soil”) and at the same time interact with circulating tumor cells (the “seeds”). This vicious, reciprocal mechanism is a crucial point in the emergence of post-surgical metastasis. According to our hypothesis, MDSCs can be the precise target to prevent cancer patients from post-surgical recurrence and metastasis during the perioperative phase to break the wretched cycle and provide better long-term survival for these patients. Future studies are needed to validate this hypothesis.

## Introduction

Myeloid-derived suppressor cells' (MDSCs) existence in pathologic conditions such as sepsis, stress, and trauma can be considered a reflection of emergency myelopoiesis. However, the tumor can utilize this phenomenon to create long-lasting abnormal myelopoiesis in favor of tumor growth and progression. Previous researches mainly focus on MDSC's role in chronic tumor environment: MDSCs can participate directly in both the adaptive and innate immune systems *via* a plethora of mechanisms, including the deprivation of arginine, the release of oxidizing molecules, the modulation of regulatory T cells (Tregs), and the interfere with T cell functions (1); and MDSC level correlates with primary tumor growth and poor prognosis (2–4).

MDSCs' function during trauma and sepsis processes has been reviewed in detail (5). In their review, Alex G Cuenca et al. believe that they may play a protective role in the host's acute stress reaction by suppressing the cytokine responses and inherent immunity. As in an acute inflammatory response process, there has been a question for quite a time: is the role of MDSC beneficial or detrimental, which has not been a satisfying answer yet. But at least the expansion in MDSCs could possibly either contributes to sepsis immune suppression or prevent it, depending on the conditions, illustrating its complexity. Ulteriorly, we are more interested in the role of MDSCs in the setting of an organism-environment where the tumor already exists.

Surgical resection is the mainstay for radically removing the primary tumor. Admittedly, surgical removal of the primary tumor is widely acknowledged as the best option in treating almost all localized solid tumors; surgery is still a significant disturbance to a living organism. Tumor recurrence and metastasis after complete resection of the primary tumor exists, resulting in a rather unsatisfactory long-term survival. Growing evidence indicates that surgery on the tumor mass can paradoxically promote post-surgical metastasis risk through complex processes that include multiple factors interplaying simultaneously (6).

Researchers have been wondering about the possible mechanism for post-surgical metastasis. MDSCs in the tumor microenvironment (TME) play a significant role in tumor metastasis (7, 8). Studies show acute stress-like surgery is likely to stimulate MDSCs growth in the TME, which then regulate the immune suppression and participate in the formation of the pre-metastatic niche (PMN) (the “soil”) (7, 9). Not only can MDSCs be induced by surgical stress, being the most obviously increased immune-related cells immediately before and after the resection of tumor lesions, post-operatively induced MDSCs are also a very potent contributor to metastases. In addition, the combination of primary tumor resection and low-dose adjuvant epigenetic modifiers or gemcitabine (which targets MDSCs) can restrain subsequent metastatic growth. This further reinforces the critical value of MDSCs in post-surgical metastasis development (8, 10). Besides their ability to forge fertile “soil” for metastasis lesions, MDSCs can also influence the fate of circulating tumor cells (CTCs) (the “seeds”).

The reasons behind post-surgical metastasis are very complicated, with metabolic, inflammatory, neural, endocrine, and immunologic factors all inseparably intertwined. We hypothesize that surgical-induced MDSCs are potent causes of post-surgical metastasis by interacting with CTCs and augmenting the PMN for CTCs to colonize and grow. In other words, MDSCs can fertilize the “soil” as well as the “seeds” at the same time. Therefore, targeting this pivotal factor and the leading source of the following cascade from surgical insult to metastasis during the perioperative period can significantly improve cancer patients' prognosis after tumor resection surgery.

## Evaluation of the Hypothesis

### Surgery Can Induce the Expansion of MDSCs

Surgery has the paradoxical capability to enhance tumor growth (11–13). Early in 1982, Uchida A has reported the possibility that circulating “suppressor monocytes” might have contributed to the inhibition of NK activity in post-operative tumor patients (14); these cells, later, were believed to be MDSCs actually. Recent endeavors have been abundant but fragmentary, spanning from inflammation, tumor cell shedding, and tumor immunity. Studies using the acute infection and sepsis model show that MDSCs increase through the expansion and activation of immature myeloid cells through the acute inflammatory process (15, 16). Surgery can also be perceived as a kind of acute stress. Evidence validates that it can induce the expansion and accumulation of MDSCs in a tumor-host, as in numerous studies in mice (17, 18) and humans (8, 19–21).

Also, the MDSCs concentration seems to correlate with the surgical procedure intensity (22, 23). In a study within breast cancer patients, research has reported that targeting the overall tumor burden through resection of the primary tumor lesions contributed to the inhibition of MDSCs, therefore promoting survival benefits (24). At the same time, there are also studies showing no significant difference in MDSC levels in different operative types, id est the surgical stress intensity (25). We have several possible explanations for this phenomenon. Firstly, the surgery itself may have reached the ceiling level of surgical stress; thus, more aggressive procedures do not necessarily result in higher MDSC-related cytokines. On the other hand, carbon dioxide (CO_2_) pneumoperitoneum could be an important factor in enhancing the metastasis-promoting ability of laparoscopic surgery (26). We suppose that besides causing peritoneal damage, CO_2_ could also facilitate tumor metastases through increasing MDSC in the local environment, as MDSC percentage increases along with the growth of arterial CO_2_ pressure (27).

Surgery possibly promotes the numerical expansion of MDSCs *via* the stimulation of the hypothalamic-pituitary-adrenal (HPA) axis and sympathetic nervous system (SNS), as well as their associated increased soluble factors and proinflammatory cytokines (IL-4, IL-10, TGF-β, and VEGF IL-6, IL-8, CXCR, CCL) (7, 28). These changes collectively create a favorable environment for the expansion and accumulation of MDSCs (29).

### Surgery-Induced MDSCs Can Augment the PMN (Soil) and Interact With CTCs (Seeds)

The previously most accepted mechanism of metastases formation is CTC being disseminated into the blood during the procedure (30). However, this is controversial since reduced or nearly unaltered CTC counts following complete tumor resection are more often observed (31, 32). Also, some researchers claim that the CTC change is not related to patient prognosis (32). Thus, tumor resection surgery promotes post-surgical metastasis, which is yet to be debated, since surgery itself does not necessarily increase the CTC numbers. Regarding this question, there is evidence showing that MDSCs can enhance the survival and metastatic function of CTCs by soluble factors as well as direct contact (9, 33). This interaction between MDSCs and CTCs is mainly composed of two aspects: direct cell-to-cell interaction and soluble factors. Firstly, MDSCs can protect CTCs in circulation from a hostile environment and facilitate their extravasation through secreting reactive oxygen species (ROS) (34, 35). Furthermore, MDSCs can directly adhere to CTCs *in vivo* and *in vitro*, form a CTC/PMN-MDSC complex, and enhance their pro-tumorigenic differentiation (36).

In addition to the interaction with CTCs, which are disseminated during the surgical procedures or discharged into the circulation before, and promote their ability to colonize and survive in the PMN, MDSCs can renovate CTCs' living conditions (PMN) as well. Surgical trauma-inflicted MDSC expansion and host immunity suppression facilitate the development of PMN (37) through releasing various MDSC-derived factors, including TGF-β, VEGF, S100A8/9, HMGB1, MMP9, TIMP-1, Arg-1, ROS, and exosomes. These factors interact as a complex network to fertile the PMN for CTCs regarding many aspects such as the colonization of CTCs, ECM remodeling, inflammation, and immunosuppressive TME (38).

Although the interference of anesthesia could confound the possible mechanisms behind the relation of surgery and post-surgical metastasis, psychological stress, surgical eradication of surrounding nerves, etc. (39–44), we hypothesize that MDSCs inflicted by surgical stress are the key players connecting these complicated mechanisms for post-surgical metastasis. In other words, MDSCs can be perceived as an orchestration of the effects of circulating cancer cells, the suppressed antitumor immunity, and the PMN of the organisms with cancer who undergo surgical resection. Thus, MDSCs should be valued as a potential target for preventing metastases from happening during the perioperative period.

## Consequences of the Hypothesis and Discussion

If the extent of surgery-induced immunosuppression manages to counteract the positive effect of primary tumor removal, surgery will fail to meet our expectations to prolong patient survival. These unwanted processes, such as MDSC expansion and its following cascade reactions, should be noted and avoided in the future. Currently, we have several methods to tackle MDSCs in cancer *via* targeting its expansion, infiltration, migration, activation, differentiation, Arg1 and iNOS induction, and so forth, which is reviewed detailedly in related reviews (45). Nevertheless, this crucial perioperative period is not given enough attention from the pharmacological intervention perspective. According to our hypothetical model, targeting MDSCs is very likely the key to preventing MDSCs induced/related post-surgical recurrence and metastasis.

**Figure 1:**
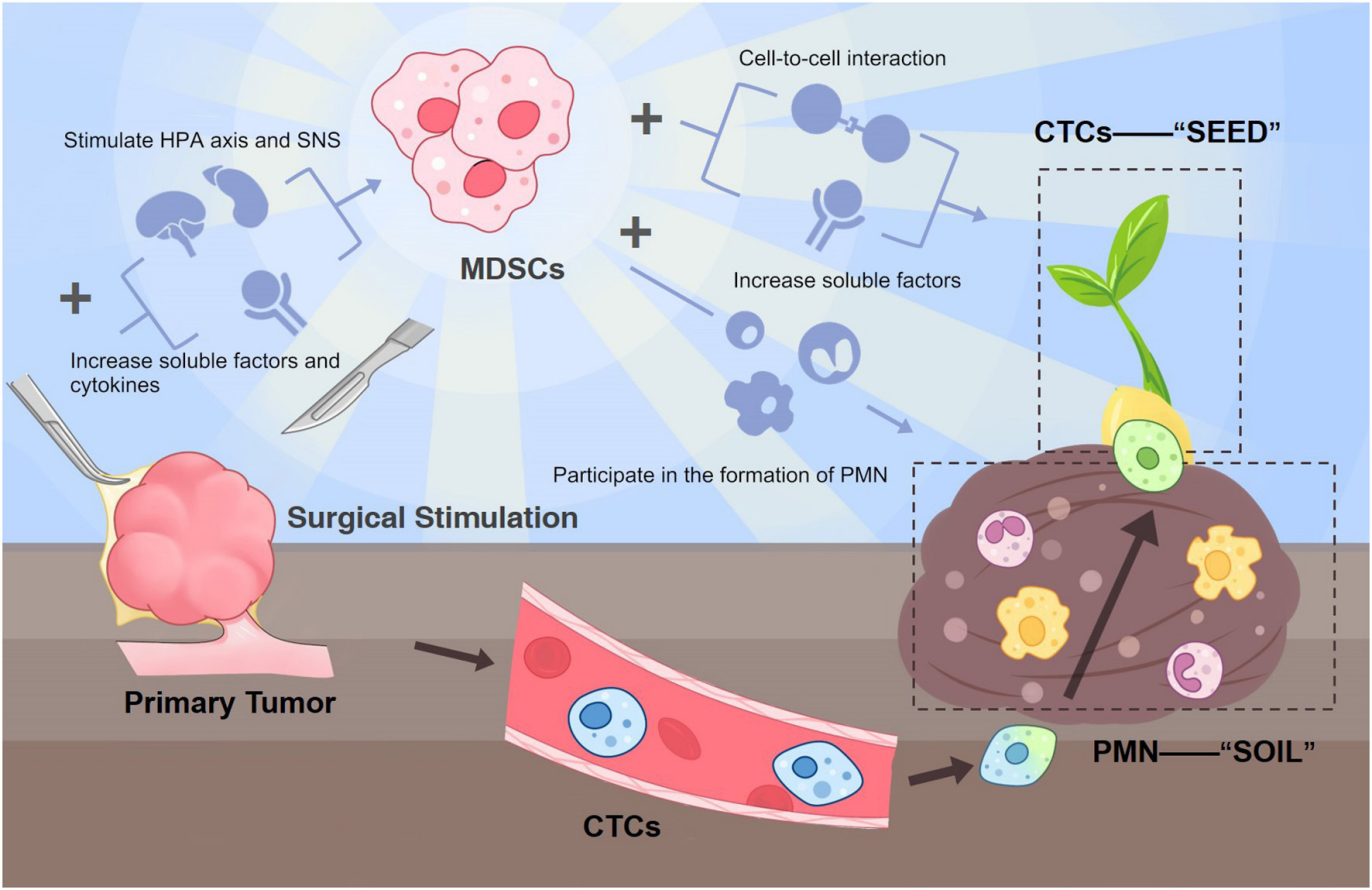
A schematic diagram of this whole hypothesis.

Future studies are encouraged to first verify the change of MDSCs in various cancer types at a different time (before and after surgery), providing a concentration curve preferably to pinpoint a more accurate window phase for future intervention. The possible existence form and structure of the MDSC-CTC complex should also be measured. *In vivo* experiments testing whether precisely removing MDSCs can reverse their effects on CTC and PMN and the following prognosis difference is also needed. Also, researchers can use flow cytometry sorting to capture CTCs and co-culture them with MDSCs extracted after emergency surgical stimulation to verify MDSC's impact on CTCs and comparing to the blank control group. Under this circumstance, when the aforementioned tests proved true, we can promisingly move on to the time when surgeons can interrupt tumor progression during the perioperative phase. A schematic diagram of this whole hypothesis is shown in Figure 1.

## Limitations

Here we propose a general model to explain what happens in the perioperative period may pre-dispose impacts on the long-time prognosis of the tumor resection procedures, mainly discussing the change and consequences of surgery-induced MDSCs. However, different primary solid tumors are likely to differ in the peripheral responses after surgery slightly, It is still needed to explore further this model in well-designed basic and clinical researches in different cancers.

## Conclusions

We hypothesize that surgical resection of the primary tumor can also facilitate the expansion of MDSCs and their pro-tumor role since these surgery-induced MDSCs can prepare the pre-metastatic niche (the “soil”) and at the same time interact with circulating tumor cells (the “seeds”). This vicious, reciprocal mechanism is a crucial point in the emergence of post-surgical metastasis. According to our hypothesis, MDSCs have the potential to be the precise target to prevent cancer patients from post-surgical recurrence and metastasis during the perioperative phase in order to break the wretched cycle and provide better long-term survival for these patients. Future studies are needed to validate this hypothesis.

## Data Availability Statement

The original contributions presented in the study are included in the article/Supplementary Material, further inquiries can be directed to the corresponding author.

## Author Contributions

SZ wrote this manuscript. YZ created the figure. XM was involved in the idea formation and manuscript revision. YQ contributed to the reviewing and manuscript editing process. All authors contributed to the article and approved the submitted version.

## Funding

This work was supported by the Natural Science Foundation of China (NSFC 81902685).

## Conflict of Interest

The authors declare that the research was conducted in the absence of any commercial or financial relationships that could be construed as a potential conflict of interest.

## Publisher's Note

All claims expressed in this article are solely those of the authors and do not necessarily represent those of their affiliated organizations, or those of the publisher, the editors and the reviewers. Any product that may be evaluated in this article, or claim that may be made by its manufacturer, is not guaranteed or endorsed by the publisher.
