# Supplementary material for: Targeting Myeloid-Derived Suppressor Cells Derived From Surgical Stress: The Key to Prevent Post-surgical Metastasis
Source: Front Surg. 2021 Dec 9;8:783218. doi: 10.3389/fsurg.2021.783218 (PMC8695559; doi:10.3389/fsurg.2021.783218)
Supplement: Supplementary file 1 [file Table_1.DOCX]

**Table 1. Summaries the main findings of key researches in this field.**

| **Author** | **Title** | **Journal, year** | **Main conclusion** | **Reference** |
| --- | --- | --- | --- | --- |
| Gabrilovich, D.I., S., et al. | *Coordinated regulation of myeloid cells by tumours.* | Nature reviews. Immunology, 2012 | Myeloid cells as an intricately connected, complex, single system and tumours manipulate the myeloid system to evade the host immune response. | 1 |
| Porembka, M.R., et al. | *Pancreatic adenocarcinoma induces bone marrow mobilization of myeloid-derived suppressor cells which promote primary tumor growth.* | Cancer immunology, immunotherapy: CII, 2012 | MDSC level correlates with primary tumor growth and poor prognosis. | 2 |
| Wang, L., et al. | *Increased myeloid-derived suppressor cells in gastric cancer correlate with cancer stage and plasma S100A8/A9 proinflammatory proteins.* | Journal of immunology (Baltimore, Md.: 1950), 2013 |  | 3 |
| Diaz-Montero, C.M., et al. | *Increased circulating myeloid-derived suppressor cells correlate with clinical cancer stage, metastatic tumor burden, and doxorubicin-cyclophosphamide chemotherapy.* | Cancer immunology, immunotherapy: CII, 2009 |  | 4 |
| Cuenca, A.G., et al. | *A paradoxical role for myeloid-derived suppressor cells in sepsis and trauma.* | Mol Med, 2011 | This review introduces MDSCs’ function during trauma and sepsis processes. | 5 |
| Benish, M., et al. | *Surgery as a double-edged sword: a clinically feasible approach to overcome the metastasis-promoting effects of surgery by blunting stress and prostaglandin responses.* | Cancers, 2010 | Surgery bears many risks leading to promotion of the metastatic process, through immune suppression, alteration of the tumor microenvironment, and direct impact on the malignant tissue. | 6 |
| Wang, J., et al. | *The influence of myeloid-derived suppressor cells on angiogenesis and tumor growth after cancer surgery.* | International journal of cancer, 2016 | Postoperatively induced MDSCs were qualified with potent proangiogenic and tumor-promotive ability and this cell population should be considered as a target for  preventing postoperative tumor metastasis | 7 |
| Lu, Z., et al. | *Epigenetic therapy inhibits metastases by disrupting premetastatic niches.* | Nature, 2020 | Even after removal of the primary tumour, MDSCs contribute to the development of premetastatic niches and settlement of residual tumour cells. A combination of low-dose adjuvant epigenetic modifiers that disrupts this premetastatic microenvironment and inhibits metastases may permit an adjuvant approach to  cancer therapy. | 8 |
| Trovato, R., et al. | *The Engagement Between MDSCs and Metastases: Partners in Crime.* | Front Oncol, 2020 | Cancer cells can establish a favorable microenvironment by manipulating the cell composition and function of the new host tissue where cancer cells can survive and outgrow, “pre-metastatic niche” (pMN), MDSCs participate in this process. | 9 |
| Bosiljcic, M., et al. | *Targeting myeloid-derived suppressor cells in combination with primary mammary tumor resection reduces metastatic growth in the lungs.* | Breast cancer research: BCR, 2019 | Targeting MDSCs and monitorting MDSC levels before and after primary tumor resection can improve the treatment of metastatic breast cancer. | 10 |
| Demicheli, R., et al. | *The effects of surgery on tumor growth: a century of investigations.* | Ann Oncol, 2008 | Surgery has the paradoxical capability to enhance tumor growth. | 11 |
| Murthy, S.M., et al. | *The influence of surgical trauma on experimental metastasis.* | Cancer, 1989 |  | 12 |
| van der Bij, G.J., et al. | *The perioperative period is an underutilized window of therapeutic opportunity in patients with colorectal cancer.* | Ann Surg, 2009 |  | 13 |
| Uchida, A., R. Kolb, and M. Micksche, | *Generation of suppressor cells for natural killer activity in cancer patients after surgery.* | J Natl Cancer Inst, 1982 | Circulating “suppressor monocytes” might have contributed to the inhibition of NK activity in postoperative tumor patients, these cells, later, were believed to be MDSCs actually. | 14 |
| Arocena, A.R., et al. | *Myeloid-derived suppressor cells are key players in the resolution of inflammation during a model of acute infection.* | European journal of immunology, 2014. | MDSCs increase through the expansion and activation of immature myeloid cells in the acute inflammatory models. | 15 |
| Schrijver, I.T., et al. | *Myeloid-Derived Suppressor Cells in Sepsis.* | Frontiers in immunology, 2019 |  | 16 |
| Pyter, L.M., et al. | *Novel rodent model of breast cancer survival with persistent anxiety-like behavior and inflammation.* | Behav Brain Res, 2017 | Surgery can induce the expansion and accumulation of MDSCs in a tumor-host in mice. | 17 |
| Ananth, A.A., et al. | *Surgical Stress Abrogates Pre-Existing Protective T Cell Mediated Anti-Tumor Immunity Leading to Postoperative Cancer Recurrence.* | PLoS One, 2016. |  | 18 |
| Li, W., et al. | *HMGB1 recruits myeloid derived suppressor cells to promote peritoneal dissemination of colon cancer after resection.* | Biochem Biophys Res Commun, 2013 | Surgery can induce the expansion and accumulation of MDSCs in a tumor-host in human. | 19 |
| Ma, X., et al. | *Myeloid-Derived Suppressor Cells Promote Metastasis in Breast Cancer After the Stress of Operative Removal of the Primary Cancer.* | Front Oncol, 2019 |  | 20 |
| Xu, P., et al. | *Surgical trauma contributes to progression of colon cancer by downregulating CXCL4 and recruiting MDSCs.* | Exp Cell Res, 2018 |  | 21 |
| Coffey, J.C., et al. | *Excisional surgery for cancer cure: therapy at a cost.* | Lancet Oncol, 2003 | MDSCs concentration seems to correlate with the surgical procedure intensity | 22 |
| Da Costa, M.L., et al. | *The effect of laparotomy and laparoscopy on the establishment of spontaneous tumor metastases.* | Surgery, 1998 |  | 23 |
| Rashid, O.M., et al. | *Resection of the primary tumor improves survival in metastatic breast cancer by reducing overall tumor burden.* | Surgery, 2013 | Targeting the overall tumor burden through resection of the primary tumor lesions contributed to the inhibition of MDSCs, therefore promoting breast cancer survival benefits | 24 |
| Buunen, M., et al. | *Survival after laparoscopic surgery versus open surgery for colon cancer: long-term outcome of a randomised clinical trial.* | Lancet Oncol, 2009 | No significant difference in MDSC levels in different operative types. | 25 |
| Gupta, A., et al. | *Tumour implantation following laparoscopy using different insufflation gases.* | ANZ journal of surgery, 2002 | CO2 pneumoperitoneum could be an important factor in enhancing the metastasis-promoting ability of laparoscopic surgery. | 26 |
| Brajer-Luftmann, B., et al. | *Myeloid‑derived suppressor cells in bronchoalveolar lavage fluid in patients with chronic obstructive pulmonary disease.* | Polskie Archiwum Medycyny Wewnetrznej, 2016 | MDSC percentage increases along with the growth of arterial CO2 pressure. | 27 |
| Kim, R. | *Effects of surgery and anesthetic choice on immunosuppression and cancer recurrence.* | Journal of translational medicine, 2018 | Surgery possibly promotes the numerical expansion of MDSCs via the stimulation of the hypothalamic-pituitary-adrenal (HPA) axis and sympathetic nervous system (SNS), as well as their associated increased soluble factors and proinflammatory cytokines (IL-4, IL-10, TGF-β, and VEGF IL-6, IL-8, CXCR, CCL). | 28 |
| Wang, J., et al. | *The influence of myeloid-derived suppressor cells on angiogenesis and tumor growth after cancer surgery.* | Int J Cancer, 2016 |  | 29 |
| Veglia, F., M. Perego, and D. Gabrilovich | *Myeloid-derived suppressor cells coming of age.* | Nature immunology, 2018 | Surgery inflicted changes create a favorable environment for the expansion and accumulation of MDSCs. | 30 |
| Yamaguchi, K., et al. | *Significant detection of circulating cancer cells in the blood by reverse transcriptase-polymerase chain reaction during colorectal cancer resection.* | Ann Surg, 2000 | Hematogenic tumor cell dissemination is a common and early event and that surgical manipulation enhances this release of tumor cells into the circulation. | 31 |
| Juratli, M.A., et al. | *In Vivo Long-Term Monitoring of Circulating Tumor Cells Fluctuation during Medical Interventions.* | PloS one, 2015 | Reduced or nearly unaltered CTC counts following complete tumor resection are often observed. | 32 |
| van Dalum, G., et al. | *Circulating tumor cells before and during follow-up after breast cancer surgery.* | International journal of oncology, 2015 |  | 33 |
| Szczerba, B.M., et al. | *Neutrophils escort circulating tumour cells to enable cell cycle progression.* | Nature, 2019 | MDSCs can enhance the survival and metastatic function of CTCs by soluble factors as well as direct contact | 34 |
| Ohl, K. and K. Tenbrock | *Reactive Oxygen Species as Regulators of MDSC-Mediated Immune Suppression.* | Front Immunol, 2018 | MDSCs can protect CTCs in circulation from a hostile environment and facilitate their extravasation through secreting reactive oxygen species. | 35 |
| Wang, L., et al. | *An Acquired Vulnerability of Drug-Resistant Melanoma with Therapeutic Potential.* | Cell, 2018 |  | 36 |
| Sprouse, M.L., et al. | *PMN-MDSCs Enhance CTC Metastatic Properties through Reciprocal Interactions via ROS/Notch/Nodal Signaling.* | Int J Mol Sci, 2019 | MDSCs can directly adhere to CTCs in vivo and in vitro, form a CTC/PMN-MDSC complex. | 37 |
| Ceelen, W., P. Pattyn, and M. Mareel | *Surgery, wound healing, and metastasis: recent insights and clinical implications.* | Critical reviews in oncology/hematology, 2014 | Surgical trauma trauma-inflicted MDSC expansion and host immunity suppression facilitate the development of PMN. | 38 |
| Wang, Y., et al. | *MDSCs: Key Criminals of Tumor Pre-metastatic Niche Formation.* | Front Immunol, 2019 | MDSC-derived factors interact as a complex network to fertile the PMN for CTCs regarding with regards to many aspects such as the colonization of CTCs, ECM remodeling, inflammation, and immunosuppressive TME. | 39 |
| Weber, M., et al. | *Macrophage polarisation changes within the time between diagnostic biopsy and tumour resection in oral squamous cell carcinomas--an immunohistochemical study.* | British journal of cancer, 2015 | The interference of anesthesia could confound the possible mechanisms behind the relation of surgery and post-surgical metastasis, psychological stress, surgical eradication of surrounding nerves, etc. | 40 |
| Yan, T., et al. | *Effects of anesthetic technique and surgery on myeloid-derived suppressor cells and prognosis in women who underwent breast cancer surgery: a prospective study.* | Cancer management and research, 2019 |  | 41 |
| Mundy-Bosse, B.L., et al. | *Psychological stress is associated with altered levels of myeloid-derived suppressor cells in breast cancer patients.* | Cellular immunology, 2011 |  | 42 |
| Koo, K.C., et al. | *Prognostic Impacts of Metastatic Site and Pain on Progression to Castrate Resistance and Mortality in Patients with Metastatic Prostate Cancer.* | Yonsei medical journal, 2015 |  | 43 |
| van den Beuken-van Everdingen, M.H.J., et al. | *Pharmacological Treatment of Pain in Cancer Patients: The Role of Adjuvant Analgesics, a Systematic Review.* | Pain practice : the official journal of World Institute of Pain, 2017 |  | 44 |
| Page, G.G. | *Surgery-induced immunosuppression and postoperative pain management.* | AACN clinical issues, 2005 |  | 45 |
| Liu, Y., et al. | *Targeting myeloid-derived suppressor cells for cancer immunotherapy.* | Cancer Immunol Immunother, 2018 | This review summarizes several methods to tackle MDSCs in cancer via targeting its expansion, infiltration, migration, activation, differentiation, Arg1 and iNOS induction, and so forth. | 46 |
